# Some Aspects of the Burning Process of Antimony and Potassium Manganate(VII) Compositions

**DOI:** 10.3390/ma15144736

**Published:** 2022-07-06

**Authors:** Marcin Gerlich, Waldemar Trzciński, Marcin Hara

**Affiliations:** 1 Faculty of New Technologies and Chemistry, Military University of Technology, Kaliskiego 2, 00-908 Warsaw, Poland; waldemar.trzcinski@wat.edu.pl (W.T.); marcin.hara@wat.edu.pl (M.H.); 2 NITROERG S.A., Alfred Nobel Square 1, 43-150 Bieruń, Poland

**Keywords:** delay composition, burn rate, gasless combustion

## Abstract

Antimony and potassium manganate(VII) compositions are widely used in time delay elements of detonators. Despite the existing literature on such systems, there is no complete information on the burning process of Sb/KMnO_4_ compositions in closed systems. There are also no data on the heat of their combustion in conditions of increased pressure without the access of oxygen from the air and on the composition of solid combustion products. These issues are the subject of the presented work.

## 1. Introduction

Sb/KMnO_4_ compositions have been the subject of many studies. Due to the widespread use of this composition as a time-delay composition, there are reports in the literature on the reaction mechanism of the thermal decomposition of potassium permanganate with subsequent antimony oxidation [1] and the burning rate of this system [2,3,4].

Based on the results of the thermal analysis presented in the literature, the combustion products should be expected to contain compounds derived from the thermal decomposition of potassium manganate(VII) and the products of antimony oxidation by the released oxygen. The occurrence of secondary reactions between the products of KMnO_4_ decomposition and the oxidation products of Sb is not confirmed. However, this possibility should not be rejected. In Ref. [1], on the basis of the DTA and TG curves, the two-stage decomposition of KMnO_4_ was confirmed and the reactions reported in the literature for the first stage of the thermal decomposition of KMnO_4_ at approx. 290 °C and, for the second stage, at 620 °C were quoted. The solid products of KMnO_4_ decomposition can be: K_2_MnO_4_, K_3_MnO_4_ and solid solutions of K_2_O and MnO_2_. According to the authors of Ref. [1], Sb_2_O_3_ and Sb_2_O_4_ are the most likely products of antimony oxidation during thermal analysis conducted in the air atmosphere. The first one occurs between 300 and 500 °C, the second one at 600 °C.

Two dominant peaks are visible on the DTA curve for the Sb/KMnO_4_ composition (with 60 wt% antimony content) heated in the air atmosphere [1]. The first one is the exothermic decomposition of KMnO_4_, which takes place at a temperature of about 300 °C. Another sharp exotherm (ignition of the composition), occurring at a temperature of about 500 °C, comes from the oxidation of Sb with oxygen from the air (as evidenced by the rapid increase in the mass of the sample in the TG curve). An important observation of the authors of Ref. [1] is the finding of a wide, exothermic peak on the DTA curve without changing the mass of the sample above the temperature of 500 °C during the test with inert gas (nitrogen). This fact is the basis for a hypothesis that, in such conditions, secondary reactions can take place in the system. On the basis of the area under the DTA curve, the total heat of the reaction for the tested composition in the air atmosphere was determined at ∆H = 2.11 kJ g^−1^. The total thermal effect of the reaction in a nitrogen atmosphere was not determined in Ref. [1].

The XRD analysis of the solid products of the combustion of Sb/KMnO_4_ compositions in the air atmosphere performed in [1] shows that in the case of high fuel content, the identified compounds were Mn_3_O_4_ and metallic Sb. For low fuel content (≤30%), the products were K_2_Mn_4_O_8_, Mn_2_O_3_, α-Mn and KMnO_2_. No crystalline antimony compounds were found.

Among other things, the burning rate of Sb/KMnO_4_ compositions with different fuel contents in the open and closed systems was tested in Ref. [3]. Antimony with two particle sizes was used in the compositions. The influence of fuel content and loading pressure on the burning rate were examined in Ref. [4].

In the available literature, there are no data on the burning products of Sb/KMnO_4_ compositions and the heat of combustion without the access of the oxygen from the air. Due to the lack of information about these products, it is impossible to estimate theoretically the heat of combustion in closed systems. From the technological point of view, the lack of reports on the influence of the density of such compositions on their burning rate is also visible. These aspects of the burning of Sb/KMnO_4_ compositions are the subject of the research presented in this paper.

## 2. Materials and Methods

### 2.1. Materials

Antimony with a purity of 99% (obtained from Stanchem Sp. z o.o. (Niemce, Poland)) and potassium manganate(VII) with a purity of 99% (obtained from STANDARD P.P.H. (Lublin, Poland)) were used to produce pyrotechnic compositions. The grain analysis of both components was performed using a FRITSCH ANALYSETTE 22 MicroTec plus laser particle size analyzer. The samples were dosed into the measuring cells by means of a vacuum dosing unit with the application of ultrasound in order to break up the agglomerates of particles. Volume-based particle size distributions were determined on the basis of laser beam diffraction. Measurements were made in the range of 0.08–2000 µm. The detected grains were divided into 102 classes in terms of their diameter (*n* = 102). The beam obscuration value was 8.0%.

### 2.2. Samples Preparation

Before mixing, the components were dried at 60 °C for three days. A TURBULA^®^ mixer was used to mix the components within 6 h. Thus, a series of Sb/KMnO_4_ compositions containing antimony in the range 30–70 wt% (with the interval of 5% (*w*/*w*) antimony) was prepared. The mass of a single batch was 50 g. Each composition was granulated (by pelleting followed by crushing using a grinder mill). Granules of a fraction of 0.2–0.8 mm were chosen for all tests.

### 2.3. Sensitivity Testing

In order to determine the sensitivity of Sb/KMnO_4_ compositions to impact, the BAM apparatus with a 5 kg hammer was used in accordance with the method given in the standard [5]. The highest energy at which no initiation was observed in six consecutive trials was assumed as the sensitivity to impact. The sensitivity to friction was determined on a Peters apparatus in accordance with the method described in the standard [6]. The greatest friction force at which no initiation was observed in six consecutive trials was assumed as the sensitivity to friction.

### 2.4. Burn Rate Measurements

The burning rate of Sb/KMnO_4_ compositions was determined on the basis of measuring the delay time of electric detonators equipped with a time-delay element with a 30 mm long column of the pyrotechnic composition. The Sb/KMnO_4_ composition was loaded in ZAMAK (ZnAl alloy) tubes with an outer diameter of 6 mm and an inner diameter of 3 mm. The compositions were dosed in 0.05 mL portions into the ZAMAK tubes and pressed on a hydraulic press. A cylindrical punch with a diameter of 2.95 mm was used for pressing. The pressure values were set using a strain gauge measuring the pressure with an accuracy of 1 N.

**Figure 1:**
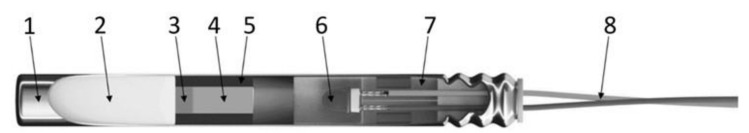
Cross-section of an electric detonator. 1—shell, 2—secondary charge, 3—primary charge, 4—time-delay composition, 5—ZAMAK tube, 6—fuse head, 7—shielding plug, 8—wire.

In order to measure the burning time of the composition pressed inside a time-delay element, electric detonators were prepared, the cross section of which is shown in Figure 1. These detonators were equipped with aluminum shells containing approx. 750 mg of secondary explosive (PETN) and a small amount of primary explosive (lead azide). The initiating system was a 0.2 A class igniter (fuse head), generating a thermal impulse of about 0.12 kJ and located at a distance of about 5 mm from the delay composition. Measurement of the delay time of such prepared detonators was based on the determination of the time between passage of the current through the fuse head and the detonation of the secondary explosive. The latter was determined by a microphone recording a sudden acoustic signal from the detonation. Obtained results were corrected by the time it took the sound wave from the detonator to reach a microphone placed at a distance of about 2 m away.

### 2.5. Heat of Combustion

Combustion heat measurements were made using the KL-12 calorimeter produced by Precyzja Bit from Poland. A cylindrical steel calorimetric bomb with a capacity of 350 mL was placed in a steel vessel containing distilled water. The calorimetric system has been described in detail in [7]. Pellets of Sb/KMnO_4_ compositions weighing from 4 to 9 g were pressed. The diameter of the pellets was 15 mm. A resistance wire made of the Kanthal D alloy (69% Fe, 22% Cr, 6% Al, 3% Zn and Si) was pressed into each pellet. The wire was 20 cm long, 2 mm in diameter and had a mass of 0.045 g. The resistance of the wire was 42.88 Ω m^−1^. The part of the wire that was pressed into the pellet was coiled into a spiral to increase its contact surface with the composition. The wire did not burn down during the tests but was broken due to the rapid increase of temperature. The heat released as a result of glow-up of the wire was not taken into account in determining the heat of combustion of the samples. The samples were placed in 4 mL quartz crucibles. Combustion heat measurements were made in an atmosphere of inert gas (argon) at various initial pressures.

### 2.6. XRD Analysis

The analysis of the crystalline structure of the solid products of combustion of Sb/KMnO_4_ compositions was performed using the Rigaku SmartLab 3 kW diffractometer with the Cu lamp (40 kV, 30 mA) and the linear detector Dtex250. The analysis of the obtained results was made using the ICDD PDF4-2020 databases and PDXL and Xrayan programs.

## 3. Results and Discussion

### 3.1. Particle Size Analysis of Antimony and Potassium Manganate (VII)

**Figure 2:**
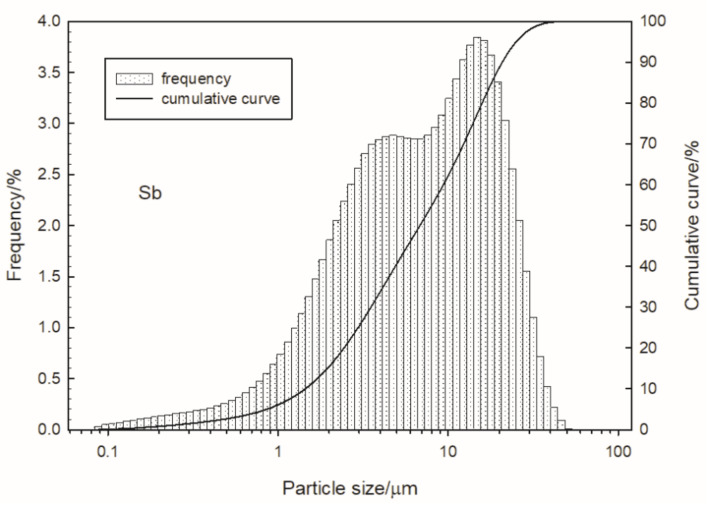
Particle size distribution of antimony.

**Figure 3:**
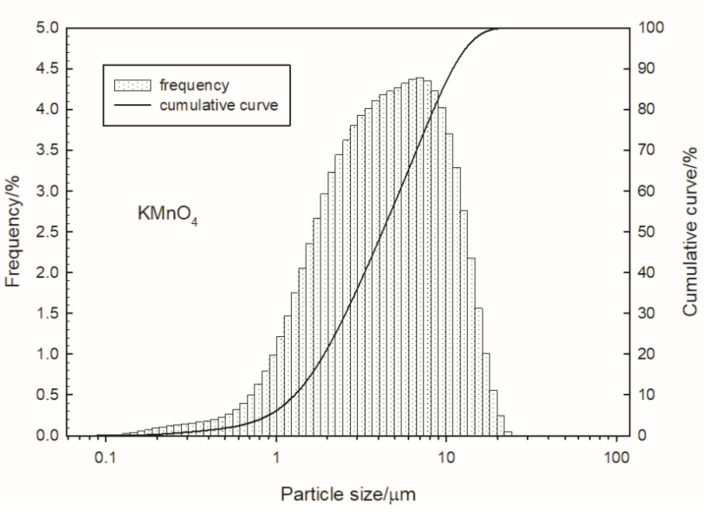
Particle size distribution of potassium manganate (VII).

Volume-based particle size distributions are shown in Figure 2 and Figure 3 for antimony and potassium manganate (VII), respectively.

Based on the measurement results, the volume-weighed moment mean (de Brouckere diameter D [3,4]) of the antimony and potassium manganate(VII) grains was determined. The de Brouckere diameter was 9.3 µm for antimony and 5.3 µm for KMnO_4_ particles. Ninety-nine percent of the total volume was occupied by the antimony particles, having a diameter (D99) of less than 34 µm; the D99 diameter was 17 µm in the case of potassium permanganate.

### 3.2. Sensitivity

The Sb/KMnO_4_ compositions were insensitive to friction (>360 N) in the entire range of the investigated antimony content. Sensitivity to impact of the tested compositions with an Sb content in the range of 30 to 60 wt% ranged from 7 to 8 J. The response of the composition significantly depended on the matching of the elements of the test system (ring and cylinder). If the system was unsealed during the positive response of the sample, the effects of the combustion of the composition in oxygen from the air (sparking and hissing) were visible. In the absence of leakage of the system, only traces of the reaction on the surface of the sample were visible, but there were no sound effects.

### 3.3. Burn Rate

**Figure 4:**
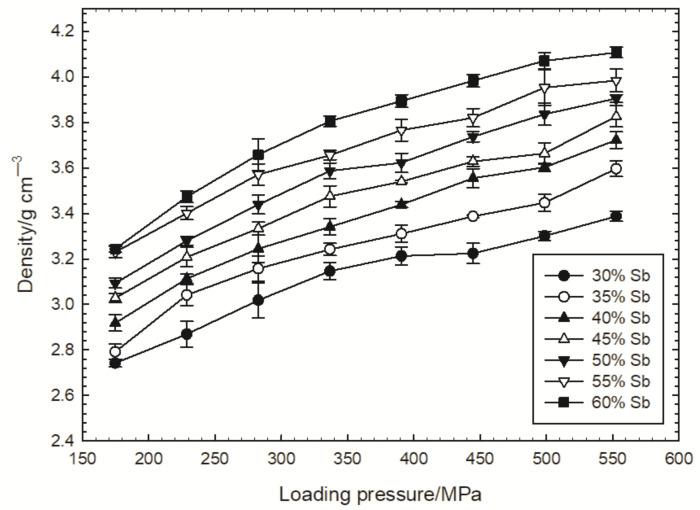
Dependence of the delay composition density on the loading pressure for different Sb contents.

For the measurement of the burning rate, delay compositions with an Sb content from 30 to 60 wt% were prepared. The burn rate of the compositions containing more than 60 wt% of the fuel was not investigated due to technological difficulties related to the granulation of the samples. The samples were loaded into tubes using different pressures. Five tests were performed for each composition and loading pressure. The dependence of the delay composition density on the loading pressure is shown in Figure 4. The figure also shows the standard deviations of the measured densities. As expected, this dependence is not linear. Generally, the increase in density decreases with the loading pressure.

**Figure 5:**
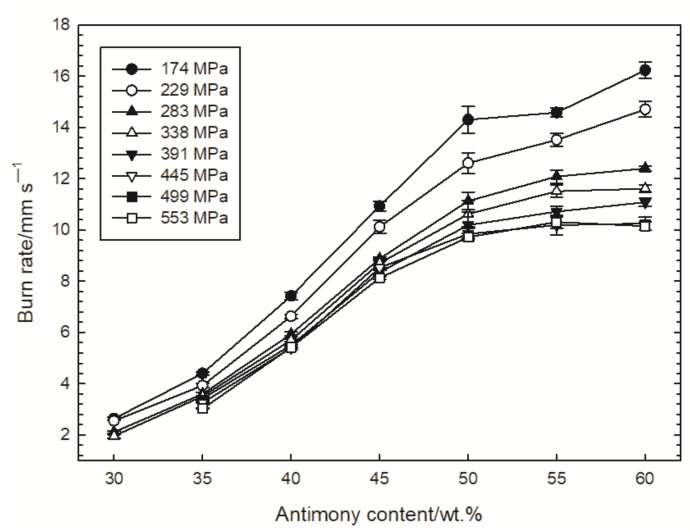
Influence of the loading pressure on the dependance of burn rate on antimony content.

**Figure 6:**
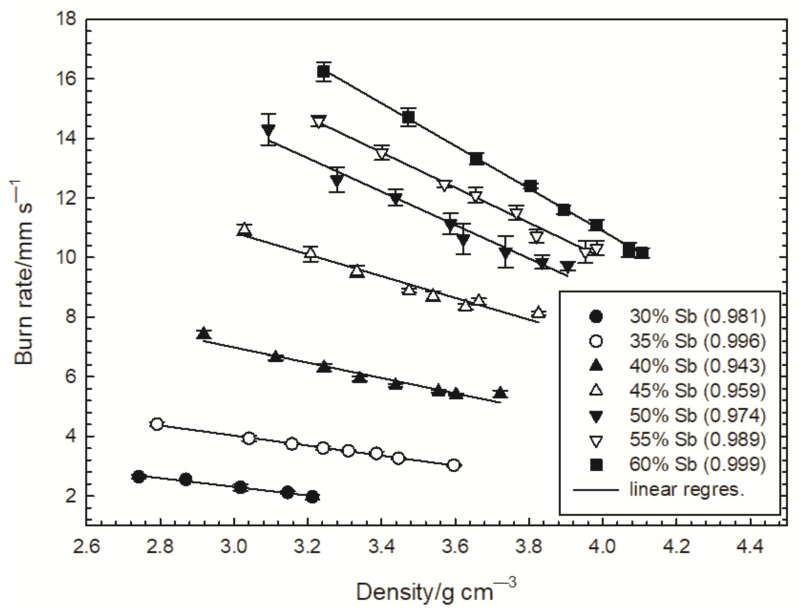
Dependence of the burning rate of tested compositions on the loading density (the correlation coefficient R2 is given in parentheses).

The dependencies of the burning rate of the tested compositions on the loading pressure and the composition density are shown in Figure 5 and Figure 6, respectively. The figures also show the standard deviations of the measured burning rates. The combustion of the Sb/KMnO_4_ composition with 30% Sb could not be initiated for a loading pressure greater than 400 MPa.

Figure 5 shows that in the tested range of the Sb content in the composition, a clear increase in the burning rate is visible. However, for a fuel content above 50%, this increase is less. These findings correlate nicely with the dependance of the delay time on the antimony content presented in [4]. The smallest changes in the dependence of the burning rate on the Sb content are visible for high loading pressures (over 400 MPa). Hence, for Sb/KMnO_4_ compositions in which more than half of the mass is fuel and which have been loaded under high pressure, no changes in the burn rate are observed. This is a great advantage of these compositions in the technology of delay detonators’ production, as it eliminates the loss of accuracy of detonators due to the possible non-homogeneous mixing of the components. Due to the high softness of the oxidant, the tested compositions can be dry granulated without the use of additional binder. However, the lack of a mixing step in the binder solution may hinder the homogenization of the production batch and necessitate the extension of the mixing process. Therefore, the occurrence of a plateau in the curves in Figure 5 is extremely important.

The dependences of the burn rate on the loading density of the Sb/KMnO_4_ composition in detonators was approximated by a linear function (Figure 6). A good correlation between the experimental data and the approximation function was obtained. The increase in the density of the composition is accompanied by a decrease in the burning rate. It is justified by the diffusion control of reactions taking place in the system, mainly antimony oxidation with gaseous oxygen resulting from the thermal decomposition of KMnO_4_. The increase in density makes it difficult for oxygen to penetrate the sample. The effect of density is especially visible for compositions with an antimony content above 45%, for which the total differences in the burning rate reach even 6 mm s^−1^. For compositions containing antimony in the range of 30–35%, the dependence of the burning rate on the density is much weaker, however, it cannot be ignored in the technology of detonators.

**Figure 7:**
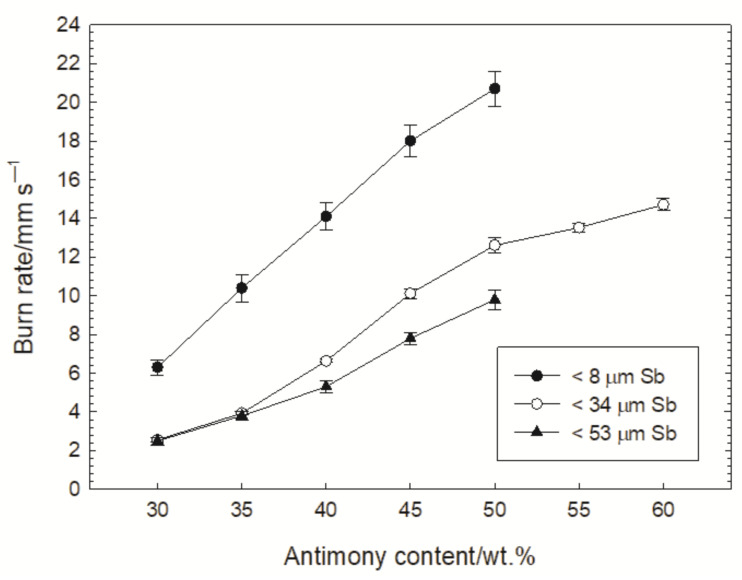
Influence of Sb particle size on the dependance of burning rate on antimony content.

It should be emphasized that apart from the quantitative composition and the loading pressure, there are also other factors that strongly affect the burning rate. Beck [2] investigated the effect of antimony grain size (<8 µm and <53 µm) on the burning rate of the Sb/KMnO_4_ composition with 30% Sb loaded with a pressure of 222 MPa into a detonator. Figure 7 presents the results given in Ref. [2] and the measured burning rates for the composition containing antimony particles with a diameter smaller than 34 µm and loaded with a pressure of 229 MPa. The determined dependence of the burning rate of this composition on the antimony content correlates well with the dependencies given in Ref. [2].

### 3.4. Heat of Combustion

In Ref. [2] pressure profiles were measured after the ignition of the Sb/KMnO_4_ compositions loaded into closed aluminum tubes. During the combustion of the Sb/KMnO_4_ compositions (with a 50 wt% antimony content), the pressure in the tube after the initial ignition pulse was constant at 1.4 MPa until the entire sample was burned, and then it gradually decreased. In the case of the compositions containing 36% and 30% Sb, the pressure initially stabilized at 2 MPa and then increased rapidly due to the evolution of oxygen and gaseous reaction products. To create combustion conditions similar to those in detonators, the calorimetric bomb was filled with argon at a pressure of 2 MPa.

**Figure 8:**
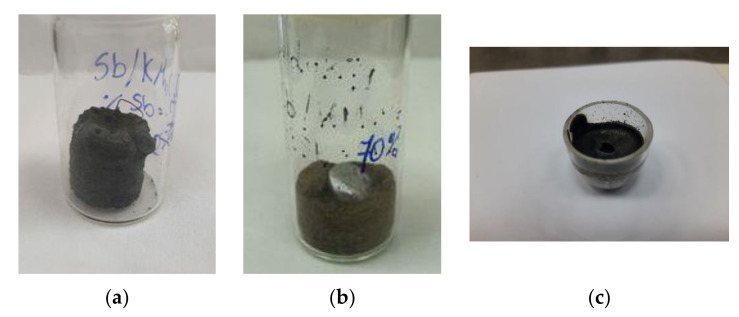
Solid products of combustion of Sb/KMnO_4_ compositions: 35% Sb (**a**), 70% Sb (**b**,**c**).

The heat of combustion of the Sb/KMnO_4_ compositions containing 30 to 70% antimony was measured. The solid products of combustion were generally shaped in a similar way as the original samples (Figure 8a), but in the case of high antimony contents, a metallic Sb ball appeared on the bottom of the quartz crucible (Figure 8b). Moreover, samples with a high antimony content melted and filled the quartz crucibles (Figure 8c).

The results of the measurement of the heat of combustion of the tested compositions are presented in Table 1. The masses of the samples before and after combustion are also given. The masses of the metallic Sb balls are shown in the parentheses. The highest values of the heat of combustion were measured for samples containing 40–45% Sb, and the lowest values were determined for samples of 65–70% Sb.

**Figure 9:**
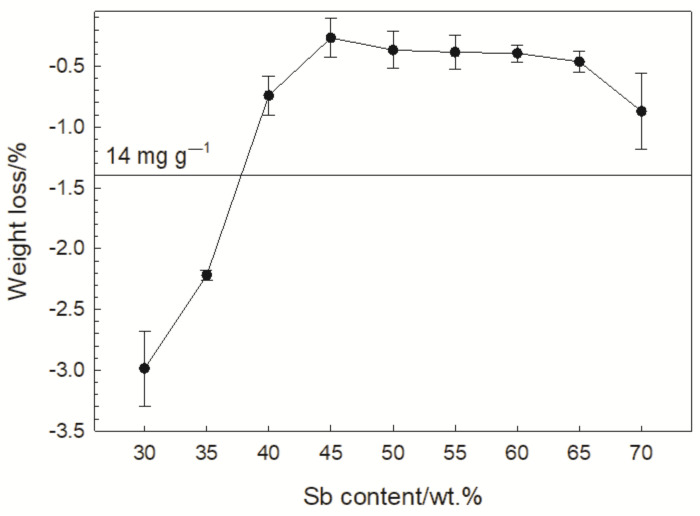
Percentage mass loss of Sb/KMnO_4_ samples burned in an argon atmosphere.

Figure 9 shows the percentage mass loss of the Sb/KMnO_4_ compositions burned in the bomb filled with argon. According to Ref. [8], the term “gasless” relates to compositions that generate up to 10 mL of gases per g of composition. In the case of the tested Sb/KMnO_4_ compositions, O_2_ may be the lightest gaseous product of combustion. A volume of 10 mL of O_2_ corresponds to a mass of approx. 14 mg. For the other gaseous combustion products of the compositions tested, the mass corresponding to 10 mL would be much higher. Therefore, it can be assumed that for the tested compositions, the criterion of 10 mL g^−1^ can be replaced with the criterion of 14 mg g^−1^. This value has been marked in Figure 9. All compositions with 40% Sb and more can certainly be treated as gasless compositions. The assessment of the compositions containing 30% and 35% Sb in this respect requires the determination of the composition of gaseous combustion products.

As the composition burns in the detonator, the pressure in the time-delay element may vary. Therefore, the combustion heat of two selected Sb/KMnO_4_ compositions for different initial pressures in a calorimetric bomb was investigated. The measurement results are presented in Table 2.

Taking into account a certain dispersion in measured heat shown in Table 1, it can be concluded from the analysis of the results presented in Table 2 that the combustion heat of the tested compositions does not depend on the argon pressure in the range of 0.2–1 MPa. Slightly higher average heat values were obtained for the pressure of 2 MPa.

### 3.5. XRD Analysis of Solid Combustion Products

**Figure 10:**
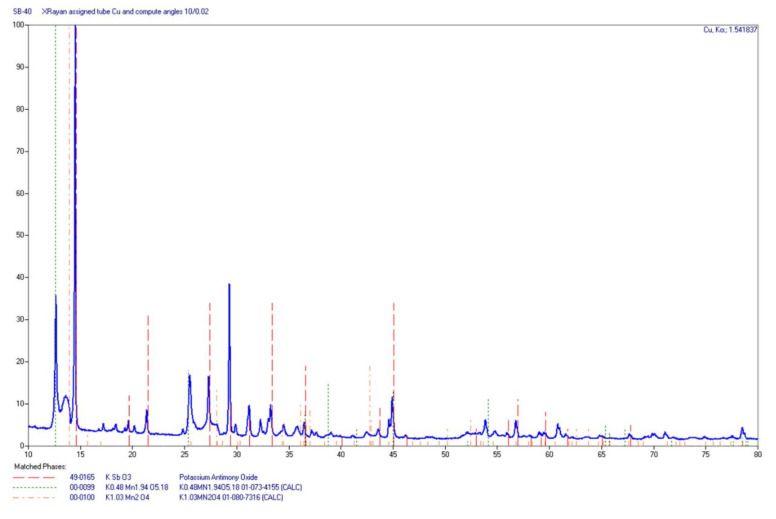
Diffractogram of the residue of the composition containing 40 wt% Sb.

**Figure 11:**
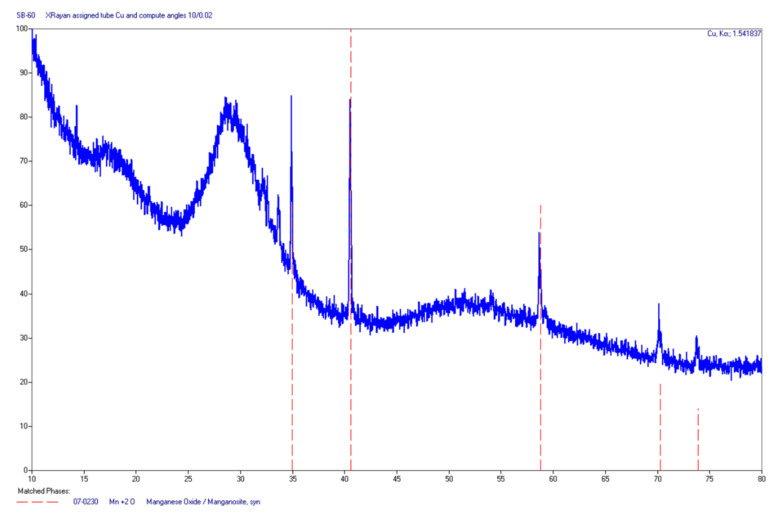
Diffractogram of the residue of the composition containing 60 wt% Sb.

The identification of the phases of a given residue after burning the Sb/KMnO_4_ sample consisted in making a powder diffractogram of such quality that reflections of a very weak intensity were distinguishable. The more well-resolved reflexes were recorded, the easier and more accurate the identification of the test substance was. Examples of the diffractograms are shown in Figure 10 and Figure 11. The results of the XRD analysis for selected samples are summarized in Table 3.

X-ray patterns show that for most of the compositions, it was not the simple antimony oxides that dominated in the reaction products, but rather potassium antymonate(V) KSbO_3_. Moreover, the analysis of the obtained diffractograms allowed us to determine the presence of non-stoichiometric K-Mn-O and K-Mn-Sb-O compounds that were not matched to the “pure” phase patterns. Unfortunately, not all phases were clearly interpreted because the degree of crystallization was not always sufficient (wide reflections). Some of the phases were solid solutions (inaccurate matching to the “clean” phase standards). Samples containing 60% and 70% Sb were mostly amorphous (Figure 11) with the local crystallization of simple oxides.

### 3.6. Thermochemical Calculations

An attempt was made to calculate the theoretical heat of the combustion of the Sb/KMnO_4_ compositions. The qualitative composition of the combustion products was determined using the CHEETAH thermochemical calculation program [9]. The CHEETAH computer program performs thermodynamic calculations for heterogeneous chemical systems containing gases, liquids or solid particles. The thermochemical equilibrium state of the reaction composition is determined by minimizing the Gibbs free energy under the condition of a mass balance of the elements present in the composition and other constraints, for example, a constant volume or constant pressure. The values of the thermodynamic functions of the individual compounds of the reacting composition necessary to perform the calculations are determined from the dependence of the specific heat *C_p_* on the temperature and the appropriate thermodynamic relationships. The dependence of *C_p_* on temperature is described by a polynomial.

(1) $$C_{p}=R\left(C_{1}+C_{2}\theta +C_{3}\theta^{2}+C_{4}\theta^{3}+C_{5}\theta^{-1}+C_{6}\theta^{-2}+C_{7}\theta^{-3}\right)$$

where *R* is the gas constant, *C*_1_, *C*_2_, …, *C*_7_ are constant coefficients, *θ* = *T*/1000 and *T* is the absolute temperature.

Before performing the calculations, the library of chemical reaction products was extended to include the potential components of the combustion products. It was not possible to find the dependence of *C_p_* on the temperature for non-stoichiometric K-Mn-O compounds, as well as some of the other compounds, which occur in solid combustion products (Table 3) or were identified as products of KMnO_4_ decomposition in Ref. [1]. However, the formation of the following gaseous substances was predicted: K, K_2_, KO, Mn, Sb, Sb_2_, Sb_4_, SbO, Sb_4_O_6_; and condensed ones: K_2_O, KO_2_, K_2_O_2_, Mn, MnO, Mn_2_O_3_, Mn_3_O_4_, MnO_2_, Sb, Sb_2_O_3_, Sb_2_O_4_, Sb_2_O_5_, MnSb, Mn_2_Sb. The temperature dependencies of specific heat and the standard enthalpy of formation of these substances necessary for the calculations were taken from the thermochemical data tables [10,11]. The constants of the relationship (Equation (1)) were determined by approximating the table data using the least squares method. The ideal gas equation was used to describe the physical properties of gaseous combustion products. Solid products were assumed to be incompressible.

In order to determine the adiabatic combustion temperature *T_a_*, it was assumed that the tested system is isolated and that the combustion process takes place under constant pressure. After determining the isobar in a given temperature range and determining the state of the thermochemical equilibrium at selected points of this range, the difference between the full enthalpy of the equilibrium state at temperature *T* and the full enthalpy of the initial composition in the standard state (*T*_0_ = 298.15 K) was calculated.

(2) $$\overset{k}{\underset{i=1}{\sum }}n_{i}{\left(I_{T}^{0}\right)}_{i}-\overset{l}{\underset{j=1}{\sum }}n_{j}{\left(I_{T_{0}}^{0}\right)}_{j}=0$$

where ${\left(I_{T}^{0}\right)}_{i}$ is the full enthalpy of the *i*-th combustion product, *n_i_* is the number of moles of the *i*-th combustion product, *k* is the number of combustion products, ${\left(I_{T_{0}}^{0}\right)}_{j}$ is the full enthalpy of the *j*-th component of the composition, *n_j_* is the moles of the *j* component and *l* is the number of pyrotechnic composition components. The full enthalpy of a substance is defined as follows.

(3) $$I_{T}^{0}=\Delta H_{f\;T_{0}}^{0}+\left[H_{T}^{0}-H_{T_{0}}^{0}\right]$$

where $\Delta H_{f\;T_{0}}^{0}$ is the enthalpy of formation at temperature *T*_0_, and $H_{T}^{0}$ and $H_{T_{0}}^{0}$ are the enthalpies of the substance at *T* and *T*_0_, respectively.

In calculations performed with the CHEETAH code, it was assumed that burning takes place under conditions of constant pressure (*p* = 2 MPa). The temperature for which the difference in Equation (2) was close to zero was treated as the adiabatic combustion temperature *T_a_*. The composition of combustion products at this temperature was the basis for determining the heat of combustion using Hess’s law. The heat of combustion was calculated as the difference between the standard enthalpy of formation of the combustion products and the standard enthalpy of formation of the initial pyrotechnic composition.

(4) $$Q_{c}=-\left[\sum_{i=1}^{k}n_{i}{\left(\Delta H_{fT_{0}}^{0}\right)}_{i}-\sum_{j=1}^{l}n_{j}{\left(\Delta H_{fT_{0}}^{0}\right)}_{j}\right]$$

**Figure 12:**
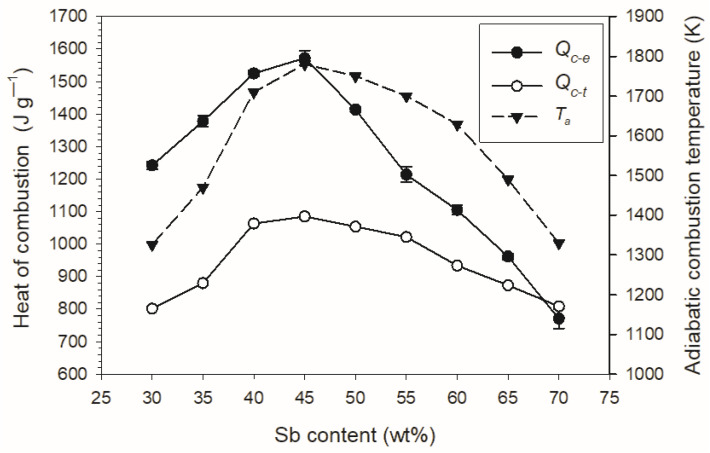
Adiabatic combustion temperature (*T_a_*), experimental (*Q_c-e_*) and theoretical (*Q_c-t_*) heat of combustion depending on the antimony content.

The dependence of the adiabatic combustion temperature and the heat of combustion on the antimony content in the Sb/KMnO_4_ composition is shown in Figure 12. The figure also shows the experimental combustion heats from Table 1.

The large difference between the calculated heat of combustion and the measured heat for almost the entire tested range of Sb content in the composition is surprising. It also means that the calculated adiabatic combustion temperature may differ significantly from the actual temperature in the combustion wave. In order to answer the question of whether the omission in the thermochemical calculations of the solid combustion products identified in the XRD analysis could be the reason for such a difference, the hypothetical combustion reactions of the 2 Sb + 2 KMnO_4_ composition were considered. The antimony content in this composition is 43.6 wt%. This value is close to the antimony content in the composition with 45 wt% Sb, for which the highest heat of combustion of 1570 J g^−1^ was obtained.

In order to calculate the heat of reaction using Hess’s law, it is necessary to know the enthalpy of formation of the substrates and products. The enthalpy of formation of the components of the 2 Sb + 2 KMnO_4_ composition and simple combustion products was taken from [10,11]. The enthalpy of formation of K_2_MnO_4_, K_2_MnO_2_, K_3_MnO_4_ and KSbO_3_ was calculated on the basis of data included in [12]. The hypothetical combustion reactions and reaction enthalpies are as follows.

(5) $$2\;{\mathrm{KMnO}}_{4}+2\;\mathrm{Sb}\;\to \;K_{2}{\mathrm{MnO}}_{4}+\mathrm{MnO}+{\mathrm{Sb}}_{2}O_{3}\;\;\;\;\Delta H_{r}=-1177\;J\;g^{-1}$$

(6) $$2\;{\mathrm{KMnO}}_{4}+2\;\mathrm{Sb}\;\to \;K_{2}O_{2}+2\;\mathrm{MnO}+{\mathrm{Sb}}_{2}O_{4}\;\;\;\;\Delta H_{r}=-977\;J\;g^{-1}$$

(7) $$2\;{\mathrm{KMnO}}_{4}+2\;\mathrm{Sb}\;\to \;K_{2}O+2\;{\mathrm{MnO}}_{2}+{\mathrm{Sb}}_{2}O_{3}\;\;\;\;\Delta H_{r}=-889\;J\;g^{-1}$$

(8) $$2\;{\mathrm{KMnO}}_{4}+2\;\mathrm{Sb}\;\to \;2/3\;K_{3}{\mathrm{MnO}}_{4}+4/3\;\mathrm{MnO}+{\mathrm{Sb}}_{2}O_{4}\;\;\;\Delta H_{r}=-1273\;\;J\;g^{-1}$$

(9) $$2\;{\mathrm{KMnO}}_{4}+2\;\mathrm{Sb}\;\to \;K_{2}{\mathrm{MnO}}_{2}+{\mathrm{MnO}}_{2}+{\mathrm{Sb}}_{2}O_{4}\;\;\;\;\Delta H_{r}=-920\;Jg{\;}^{-1}$$

(10) $$2\;{\mathrm{KMnO}}_{4}+2\;\mathrm{Sb}\;\to \;\;2\;{\mathrm{KSbO}}_{3}+2\;\mathrm{MnO}\;\;\;\;\Delta H_{r}=-1733\;J\;g^{-1}$$

The assumption of different reaction products means that the heat of combustion can differ even twice. The lowest thermal effects are obtained by assuming only simple metal oxides in the products (Equations (6) and (7)). Similarly, the presence of simple oxides is assumed in thermochemical calculations using the CHEETAH code. For example, for a composition containing 45% Sb, the calculated combustion products are the solids K_2_O, Mn_3_O_4_, Sb_2_O_4_ and small amounts of O_2_, K and KO gases. The calculated heat of combustion for this composition is 1085 J g^−1^. The measured value is much higher (1570 J g^−1^). However, the XRD analysis shows that KSbO_3_ is the dominant component of the combustion products of compositions with a similar composition (40% Sb—Table 3). Its presence in reaction products significantly increases the heat effect (Equation (10)). Thus, the reason for the higher values of the measured heat of combustion of the tested compositions is the presence of KSbO_3_ and non-stoichiometric K-Mn-O compounds. The above analysis shows that the only way to determine the heat of combustion for Sb/KMnO_4_ compositions are calorimetric measurements in an inert gas. The heat determined in this way can be used in modeling the combustion process of these compositions, for example, with the use of models [13,14,15].

## 4. Conclusions

The results of the research on the burn rate of the two-component Sb/KMnO_4_ composition as a function of the antimony content and the composition density are presented. The system containing antimony with a grain diameter of <34 µm, pressed in ZAMAK (ZnAl alloy) tubes, burned at a rate of 2 to 16 mm s^−1^. An increase in the antimony content caused an increase in the burning rate. The inverse relationship was obtained in the case of the composition density—an increase in the composition density was accompanied by a linear decrease in the burning rate.

The heats of combustion of Sb/KMnO_4_ compositions were determined in a calorimetric bomb filled with argon at a pressure close to the pressure in detonators with such delay compositions. For the Sb content in the composition from 30 to 60 wt%, the measured heats of combustion were much higher than the heats calculated using the thermochemical code, in which only simple metal oxides were considered as potential reaction products. At the same time, XRD analysis showed that such oxides are not identified in solid combustion products, but rather KSbO_3_ and non-stoichiometric K-Mn-O and K-Mn-Sb-O compounds. Consideration of the hypothetical reactions for one of the compositions in which, in addition to simple metal oxides, KSbO_3_ and potassium manganates with manganese at lower oxidation states were included, showed that the heat effect is significantly greater.

The presented research results extend the literature data to include the heat of combustion of Sb/KMnO_4_ compositions in a wide range of antimony contents and the combustion rate of this system as a function of the antimony content and composition density. These results constitute the first step to developing a model of Sb/KMnO_4_ composition burning in time-delay elements.

## Figures and Tables

**Table 1:** Results of combustion of Sb/KMnO_4_ compositions in the calorimetric bomb filled with argon at a pressure of 2 MPa (the mass of the metallic Sb in parentheses).

Sb/KMnO_4_ [wt%/wt%]	Sample Mass [g]	Residue Mass [g]	Combustion Heat [J g^−1^]	Average Combustion Heat [J g^−1^]
30/70	4.9040	4.7734	1252	1240
	4.4400	4.2932	1231	
35/65	6.9920	6.8342	1367	1380
	7.2570	7.0989	1388	
40/60	7.2415	7.1766	1507	1525
	6.8123	6.7725	1542	
45/55	7.2903	7.2824	1594	1570
	5.5263	5.5028	1549	
50/50	7.8328	7.8163	1421	1410
	5.3226	5.2949	1406	
55/45	8.5813	8.5602 (0.0295)	1238	1210
	6.2187	6.1862 (0.0372)	1191	
60/40	8.5718	8.5390 (0.6443)	1119	1105
	9.4184	9.3814 (0.7904)	1090	
65/35	9.2868	9.2440 (1.7131)	972	960
	5.9650	5.9374 (1.1033)	952	
70/30	8.5049	8.4018 (2.7425)	801	770

**Table 2:** Influence of argon pressure on the heat of combustion for selected Sb/KMnO_4_ compositions.

Argon Pressure [MPa]	Heat of Combustion [J g^−1^]	
	40 wt% Sb/KMnO_4_	60 wt% Sb/KMnO_4_
2	1507	1119
	1542	1090
1	1473	1080
0.5	1506	1061
0.2	1458	1087

**Table 3:** Solid products of combustion identified by XRD analysis.

Sb Content [wt%]	Compounds	
	Argon at a Pressure of 2 MPa	Argon at a Pressure of 0.2 MPa
30	KSbO_3_, Mn_3_O_4_, K_2·x_Mn_8_O_16_	-
35	KSbO_3_, K_0.48_Mn_1.94_O_5.18_, K_1.03_Mn_2_O_4_, K_0.68_Mn_0.56_Sb_0.44_O_2_	-
40	KSbO_3_, K_0.48_Mn_1.94_O_5.18_, K_1.03_Mn_2_O_4_	KSbO_3_, K_2_Mn_4_O_8_, K_0.68_Mn_0.56_Sb_0.44_O_2_
50	KSbO_3_, K_2_Sb_2_O_7_, Mn_3_O_4_,	-
60	MnO, Sb	MnO, Sb
70	MnO, Sb, Sb_2_O_3_	-
